# Obituary

**Published:** 1889-03

**Authors:** 


### OBITUARY.

In our last issue we called attention to the serious illness of Dr. Flavius Searle, of Springfield, Mass., which resulted fatally on February 10, 1889. Dr. Searle was born in Southampton, where he received his early education. He was the oldest dentist in Springfield. He learned dentistry as a trade, but lived long enough to see it exalted to a profession, and had the satisfaction of knowing that he had contributed not a little to that end.

Dr. Searle was recognized as one of the leading men in his profession. He was a charter member of the Connecticut Valley Dental Association, of which he was the first president, and a member of many other organizations of the kind. He belonged to the wide-awake school of progressive dentistry, and always welcomed and practiced any modern and useful advance in the science. He was a close student, spending his spare moments in study and research, and he never tired of doing what he could toward advancing the professiion he loved so well. In 1887 the Connecticut Valley Dental Association tendered Dr. Searle a banquet in honor of his rounding out of 50 years of life as a dentist. The entertainment was an elaborate one, and brought letters and other evidences of good-will from prominent dentists all over the country. A record of the proceedings was published in the form of a memorial volume, elegantly bound, and presented to Dr. Searle as a memento of the occasion, a description of which was published in this journal last year.

Dr. Searle married Miss Abigail Brown, of Brimfield, and had three children, all daughters. His wife died about six years ago. Two years later his eldest daughter passed away, while a second daughter died in childhood. His only living daughter now resides in Boston.

Dr. Searle will long be remembered as the friend of the young practitioner, an earnest seeker after truth, and a man who had the courage of his own convictions, and who was not afraid to speak them when occasion required.

---

In the death of Dr. Brasseur, dentistry loses one of its leading members, the *International Dental Congress* one its most active promulgators and our Journal a valued correspondent. We had not the pleasure of an acquaintance other than by correspondence, but our intercourse with him had been of the pleasantest character and bade fair to become profitable. We have asked Dr. Bogue, who was intimately acquainted with Dr. Brasseur, to send us some points relating to his life and demise, and which we have incorporated into this notice.

"Our profession has lost in France a most valuable member by death, Dr. Emile J. N. Brasseur. He was to have dined with a number of professional brethren on the 22d of January. He sent word at seven o'clock not to wait for him, as he was ill. He died about the time the meeting broke up.

Dr. Brasseur was a graduate of the Medical College of France, and he was at the time of his death the President of the Odontological Society of France, the Director of the Dental School of the Rue de l'Abbaye, the Editor of the Revue Odontologique, and he had just accepted the post of correspondent of the "INTERNATIONAL."

He sent the best illustrated article that came to the dental section of the International Medical Congress on the "Therapeutic Use of Hot Air."

A hot air injector was one of his inventions; so also was an electric examination light combined with an exploring point; an instrument for compressing six or eight amalgam blocks at once was his last invention. It was he who wrote the article on "Dental Surgery" in the new French Encyclopædia.

Wherever work was to be done for the advancement and upbuilding of the dental profession, Dr. Brasseur was to be found doing his share.

He recognized no nationality in science.

He was a friend of Telschow, the German dental inventor.

Rosenthal, the Belgian, exhibited his injector at Dr. Brasseur's office.

My first meeting with him was through Sir John Tomes, and for us Americans he has shown the warmest friendship at all times.

His death leaves a void almost impossible to fill, and those of us who knew him must deeply mourn his loss."

